# Patient-specific 3D-bioprinted anti-fibrosis barriers to reduce adhesions after abdominal and pelvic surgery

**DOI:** 10.1097/JS9.0000000000004738

**Published:** 2026-01-07

**Authors:** Sarim Hassan Shahab, Aoun Hassan, Fazal Habib

**Affiliations:** a Department of Internal Medicine, Nishtar Medical University, Multan, Pakistan; b Department of General Surgery, Services Institute of Medical Sciences, Lahore, Pakistan; c Department of Internal Medicine, Sher-e-Bangla Medical College, Barisal, Bangladesh

## Abstract

Adhesions after surgery continue to pose serious issues. Postoperatively, adhesions are one of the most common causes of chronic abdominal pain, infertility, intestinal blockage, and healthcare-related financial burden. Only a few anti-adhesion products have been developed to prevent these problems and most of them show suboptimal outcomes due to a lack of conformity to the location of the surgery, poor durability, and low bioactivity. In this letter, we discuss the need for new solutions, and present a new concept: Customized, 3D-printed barriers (Bio-Printed Anti-Fibrosis Barriers) made from regenerative biomaterials. Custom-designed biomaterials can fit precisely into the surgical cavity, provide focused delivery of anti-inflammatory agents, and guide the regeneration of tissue. The convergence of bioprinting technology and regenerative biomaterials may serve as an innovative avenue for decreasing morbidity associated with adhesions after oncologic and reconstructive surgery.

Postoperative adhesions are one of the most common and challenging complications for surgeons globally. Even with the advancement of minimally invasive techniques, postoperative adhesions are common; they may range from 50% to 100% in case of abdominal surgery and 60%–90% after gynecologic procedures, leading to chronic pain, sub-fertility, intestinal obstruction, and expensive reoperation^[1,2]^. Patients frequently experience a good recovery from their initial surgical procedure, but months or years later return with persistent complications related to adhesions^[3]^. This situation shows an unaddressed need for new therapeutic methods to prevent the formation of postoperative adhesions. The products used to prevent the formation of postoperative adhesions remain limited, inconsistent, and biologically passive.

A variety of current products exist to prevent postoperative adhesions, including: gels made from hyaluronic acid; polymers made from collagen; synthetic membranes made of various polymer types (biodegradable and non-biodegradable), etc^[4]^. These current anti-adhesion products have resulted in incremental improvements, but continue to be deficient in three main areas: they degrade too quickly to provide ongoing support for wound healing; they may carry the risk for post-operative complications including abscess, infection, anastomosis leakage and fistula; they do not contain regenerating or antifibrotic agents which promote tissue regeneration^[5–7]^. Most importantly, they are “one size fits all” products, not taking into account the inherent variability of patients with respect to the inflammatory response, wound healing, and the geometry of the patient’s tissue to be treated. In oncological surgery, where large and complex resection sites are common, the limitations associated with the currently marketed products are magnified.

The need to rethink how we prevent adhesions has never been more urgent. We believe that the technology of 3D bioprinting represents a very good opportunity for the creation of engineered barriers against fibrosis that are patient-specific for the next generation. The unique precision and adaptiveness of bioprinting would allow each construct created by laboratories to be perfectly placed to the surgical site with a uniform level of coverage that cannot be achieved with existing methods such as biomembranes^[8]^. A concise comparison between current anti adhesion barriers and proposed 3D-bioprinted barriers in described in Table 1.

**Table 1:** A concise comparison highlighting the potential advantages of 3D-bioprinted adhesion barriers.

Feature	Current anti-adhesion products	3D-bioprinted barriers	Clinical implication
Anatomical fit	One-size-fits-all; poor conformity to irregular surgical cavities	Patient-specific constructs designed from imaging data	Improved coverage reduces adhesion recurrence risk
Bioactivity	Mostly passive physical barriers; minimal regenerative/anti-fibrotic properties	Bioactive bioinks with cells, hydrogels, growth factors, cytokine release	Actively modulates healing and fibrosis pathways
Durability & degradation	Often degrade too quickly or unpredictably	Tunable degradation matched to healing timeline	Maintains protection during the critical fibrotic phase
Complication profile	Risk of infection, abscess, inflammatory irritation, or anastomotic leakage	Designed to reduce inflammation, with controlled drug loading	Potentially lowers postoperative complications
Customization & versatility	Limited options; cannot adapt to patient variability	Fully customizable for defect size, shape, pathology, and surgical type	Enables precision medicine in abdominal/gynecologic/oncologic surgery

By creating bioinks with regenerative elements such as mesenchymal precursor cells, anti-fibrotic hydrogels, and sustained-release anti-inflammatory cytokine treatments, the bioprinted construct may actively modify the healing environment, as opposed to being limited to a passive separation that only provides a general barrier against the formation of adhesions. These constructs would serve to limit the excess activity of fibroblasts, control excessive deposition of collagen, and support the restoration of an organized, non-scarred healing process^[9,10]^.

Because 3D printing technologies are flexible, a surgeon can continue using bioprinting after surgery for many different purposes. For example, if the surgeon has obtained a pre-surgical image that shows where they would like to place their adhesive, they would be able to use the printer to make a custom adhesive barrier for that location. Although the workflow described above needs further confirmation, the bioprinting technology used to create this workflow is already known in the field of regenerative medicine.

Incorporating bioprinting into surgical procedures, such as preventing postoperative adhesion formation, represents a significant advancement and one that can be implemented within a short period of time. While the concept of bioprinting an entire organ is a future possibility, this type of application has the potential to be of immediate use in helping the recovery of patients following complex surgical procedures, such as those associated with oncologic and reconstructive surgery. Clinical trials along with robust ethical, technical, and regulatory frameworks will be essential for ensuring patient safety, standardize procedures and facilitate clinical adoption of these bioprinted constructs.

Thank you for considering this perspective. We hope it inspires further discussion and research toward more personalized, biologically intelligent post-surgical solutions.

Note: This letter complies with TITAN-2025 guidelines and checklist^[11]^.

## Data Availability

All data and materials used in this editorial are derived from publicly accessible sources, including peer-reviewed articles and referenced scientific literature. Full citations are provided within the text to ensure transparency and facilitate further exploration.
